# The Pure Milk Problem

**Published:** 1918-12-14

**Authors:** 


### THE PURE MILK PROBLEM.

If bread be the staff of life, milk may be called the crutch, for it is the main prop both of the child and of the invalid. It is the diet *par excellence* of the nursery and of the hospital. The unfortunate thing is that it is also the favourite food of innumerable microbes, which not only batten on it but bathe in it and breed in it, so that unless certain precautions are taken, the child and the invalid drink not only milk, but also a few million microbes and various microbial products. The microbes, too, are not infrequently pathogenic, and the products not infrequently poisonous. The milk-can, in fact, is often little better than an incubator of germs, and the dairy purveys not only milk but enteric fever and other germ diseases.

### The Significance of Micro-Organisms.

We are not much impressed by sensational and alarming figures showing how many million living organisms may swarm in a cubic centimetre of milk. A pot may not call a kettle black, and we whose intestines in health harbour microbes by the billion, can hardly refuse hospitality to a few million more, especially since most of the microbes in milk are probably as harmless as the currants in buns, and some, such as the lactic acid bacillus, even philanthropic. It is not a question of man power; it is not a question simply of numbers. It does not matter very much whether there are a million or ten million micro-organisms per cubic centimetre. The point is simply this, that if milk be swarming with micro-organisms (even benevolent or neutral ones) it is dangerous; for the micro-organisms are signs and symptoms of dirty and careless dairy methods, and are an index of the opportunities of access and growth offered to germs of disease. If the conditions of milking and carriage have allowed the entrance and multiplication of myriads of harmless germs, then there has obviously been every chance for hostile germs to enter and multiply too.

### Some Alarming Figures.

That, indeed, is quite alarming enough, even if we recognise that millions of microbes in the milk may not in themselves be quite so deadly as they sound. But much more alarming, because much more precise, are figures showing how many samples of milk do actually contain definite disease germs. Of such figures the most certain are those which refer to tuberculosis, and when we learn that 10 or 12 per cent. of milk comes from tuberculous cows and contains the tubercle bacillus, that is surely enough to waken the hygienic conscience of everybody.

### Milk Kills more Men than Bullets.

We do not exaggerate when we say that milk has killed more men than bullets have ever laid low. Not only has it killed, but it is still killing; still the casualty list, still the roll of dishonour is mounting up. Still dirty and dangerous milk is being supplied to the English nation.

### Disgrace and Criminal Neglect.

It is nothing less than a disgrace that in many towns 10 per cent. of the milk comes from tuberculous cows. It is a disgrace, and it is high time that strong measures were taken against such a state of affairs. All that is required is healthy cows, good byres, with intelligent supervision, and if these requirements mean that milk must cost a little more, the price is worth paying. Surely it is better to pay a few pennies more a gallon and get pure milk than pay a few pennies less and run the risk of doctors' fees and undertakers' bills.

Nor should choice be possible. There should be no competition between pure milk and impure milk. One of the first things a Ministry of Health must see to is that *all* milk be pure milk from healthy cows. Meantime hospitals and public institutions ought to lead the way. It is amazing to find that many hospitals and institutions do not at present take due precautions to obtain pure milk. It is very strange that any hospital or public institution should be so short-sighted, and false to their trust, as to save money at the risk of losing life.

